# Antiretroviral activity of *Pterois volitans* (red lionfish) venom in the early development of human immunodeficiency virus/acquired immunodeficiency syndrome antiretroviral alternative source

**DOI:** 10.14202/vetworld.2019.309-315

**Published:** 2019-02-23

**Authors:** Andy Noorsaman Sommeng, R. Muhammad Yusuf Arya, Mikael Januardi Ginting, Diah Kartika Pratami, Heri Hermansyah, Muhamad Sahlan, Anondho Wijanarko

**Affiliations:** 1 Department of Chemical Engineering, Faculty of Engineering, Universitas Indonesia, Indonesia; 2 Marine Science Postgraduate Program, Faculty of Mathematics and Natural Sciences, Universitas Indonesia, Indonesia; 3 Laboratory of Pharmacognosy and Phytochemistry, Faculty of Pharmacy, Pancasila University, Indonesia; 4 Research Center for Biomedical Engineering, Faculty of Engineering, Universitas Indonesia, Indonesia

**Keywords:** antiretrovirus, lamivudine, phospholipase A2, *Pterois volitans*, simian retrovirus serotype 2

## Abstract

**Aim:**

This study aimed to investigate the antiviral activity of *Pterois volitans* phospholipase A2 (PV-PLA2) from Indonesia to human immunodeficiency virus (HIV).

**Materials and Methods:**

Fresh venomous fin parts of wild PV specimens were collected from Java Sea waters. Then, it washed using phosphate buffer pH 7.0 and immersed in phosphate buffer pH 7.0 0.01 m containing CaCl_2_ 0.001 m for 24 h. The immersed fin then allowed for extraction process by sonicating for 2×8 min with 80% pulse and 20 kHz output with temperature controlling to avoid denaturation. The crude venom (CV) extracted from the fin is allowed for purification by 80% ethanol (ET) precipitation and ammonium sulfate fractionation method. The purified PV-PLA2 then analyzed using Lowry’s method, Marinette’s method, sodium dodecyl sulfate-polyacrylamide gel electrophoresis, and 3-(4, 5-dimethyl thiazol-2yl)-2, 5-diphenyl tetrazolium bromide assay. After determining the purest and safest sample of six samples analyzed, the chosen sample then tested into simian retrovirus-2 (SRV2)-A549 culture (48×10^4^ cells/mL at 1-4 ppm), and compared to the CV sample (1-4 ppm) and lamivudine (100 ppm). The culture then is analyzed using a quantitative real time-polymerase chain reaction to find out the copy number of SRV-2 virus in each culture.

**Results:**

The protein’s activity, concentration, and purity analysis revealed that the PV-PLA2 purified using ammonium sulfate fractionation has the highest activity (1.81 times higher than the CV at 80% fractionation) and has higher purity than the sample from ET fractionation. The testing of the sample purified using ammonium sulfate fractionation at 80% saturation level shown that it has a 97.78% inhibition level toward SRV2-A549 culture at 4 ppm. However, in comparison to lamivudine which has 99.55% inhibition level at 100 ppm, it needs much lower concentration to achieve the same result.

**Conclusion:**

The significant inhibition of SRV2-A549 culture shown that the PV-PLA2 extracted from PV venom has the potential to become anti-HIV substances. It would be worthwhile to further evaluate the antiretroviral activity of PV-PLA2 in the *in vivo* studies.

## Introduction

Red lionfish (*Pterois volitans* [PV]) is red and white striped fish that have native habitats in the Indo-Pacific Ocean [1]. Red lionfish - a nocturnal predator species that consume crustaceans, small fish, and crabs - is an invasive species that attacks in new waters to obtain food [2]. In the past 8 years, native red lionfish from Indo-Pacific Sea has spread to the Atlantic Ocean in the Bahamas, Caribbean [3]. The absence of natural predators in the Atlantic Ocean causes the red lionfish to breed rapidly and reduce the native fish population by up to 80% [4]. Furthermore, invasion attacks by red lionfish can cause ecosystem imbalances by disrupting the food chain and damaging coral reefs that cause a drastic change in the population [5,6]. PV is a threat to Indonesia’s marine biodiversity. The native habitat of these animals is the Indo-Pacific Ocean adjacent to the Indian Ocean. In addition to being invasive, it is also a poisonous animal having neurotoxins in the thorns to protect itself from predators [7]. These animals do not have natural predators to control their population. In Indonesia, poisons in the body of red lionfish have the potential to be utilized in pharmaceuticals. PV is closely related to *Pterois russelii* or Persian lionfish which has venom that contains phospholipase A2 (PLA2) [7].

PLA2 is a protein compound that has antiretroviral activity against human immunodeficiency virus (HIV) [8-10]. The mechanism of action of PLA2 against HIV based on fact *Acanthaster planci*-PLA2-I and -II have hemolytic activity only in the presence of phosphatidylcholine (PC), which releases fatty acids that act as antibacterial agents and also possess myotoxic activity [11,12]. It is expected that red lionfish also has the same protein (PLA2) content. Acquired immunodeficiency syndrome (AIDS) is a disease caused by HIV, which can be transmitted through one or more of the high-risk behaviors, such as sharing needles in injecting drug users, unsanitary commercial blood donation, unprotected sexual intercourse, and mother-to-child transmission [13-15]. The estimated number of people living with HIV (PLWH) in Indonesia has increased significantly during 2004-2015 (5846-690,000), and the number of AIDS patient diagnoses has also increased during 2004-2015 (4973-77,112) [15]. The Indonesian government has been distributed free of charge antiretroviral therapies (ARTs) since 2004. The ART had successfully reduced the HIV-related mortality rate. The fatality rate was dramatically declined from 2004 to 2015 [15]. The need for ART in Indonesia is expected to decrease in HIV-AIDS patients. However, only 24% of PLWH in Indonesia who are eligible for treatment receive ART due to constrained medical budget for HIV/AIDS control [16].

Based on this information, the present research explores the characteristics of PV-PLA2 and assesses its potential as a natural, cheap, safe, and environmentally friendly ART drug for the treatment of HIV infection. This study aimed to investigate the antiviral activity of PV-PLA2 from Indonesia to HIV. An extraction method, to obtain the content of PV-PLA2, is needed which can extract the enzyme content from the venom and maintain the stability of the enzyme so that it can be used directly. The effective enzyme extraction method can be used with a sonicator to extract toxins from solids with the help of pH 7.0 phosphate buffer solution [17]. With this method, the content of PLA2 can be obtained in high concentration at a relatively cheaper cost. It is expected that using this method, ART drugs can be produced at lower prices.

## Materials and Methods

### Ethical approval

This *in vitro* study does not need ethical approval from the University Ethics Committee.

### PV stings’ preparation and venom extraction

The pre-treatment was carried out based on the standardized method reported by Ibrahim *et al*. on *Acanthaster planci* sea star spines [11]. The separation of the stings from the body of the PV from Java Sea waters has done by cutting it from the base in cooler conditions. The stings that have been successfully separated are then rinsed with 0.01 m phosphate buffer pH 7.0.

Extraction was carried out using sonication with pre-treatment by soaking the thorn fins in a solution of 0.01 m phosphate buffer pH 7.0 containing 0.001 m CaCl_2_. 50 g of stings were soaked in 100 ml of buffer solution. Then, sonication was carried out for 2×8 min with an 80% pulse and an output of 10 at 20 kHz and maintained cold temperatures during the process. Later on, centrifugation is done to separate the impurities such as fine particles and denatured proteins. Venoms extracted and dissolved in a buffer solution are called crude venom (CV).

### PLA2 purifications

There are two methods of purification carried out, namely ethanol (ET) precipitation and ammonium sulfate fractionation. In ET precipitation, CV is first filtered using a vacuum filter to purify CV from solid impurities such as connective tissue and denatured protein. Furthermore, the obtained filtrate was dissolved in 99.9% analytical ET with 80% ET concentration or 1:4 volume ratio between venom and ET. The mixture was incubated at a temperature in the 4°C for 24 h in a closed container. The precipitate in the form of the suspension formed was separated from the mixture by the decantation process and then centrifuged at 4500 rpm for 30 min. The precipitate formed was the PV-PLA2 fraction in the process which was separated and redissolved using a 0.01 m phosphate buffer solution pH 7.0.

Fractionation with ammonium sulfate was carried out by adding ammonium sulfate little by little in CV with a saturation level of 20%, 40%, 60%, and 80% with a magnetic stirrer for approximately 20 min. Each sediment of enzyme protein obtained was separated from the filtrate using centrifugation at 4500 rpm for 30 min. The precipitate formed was the PV-PLA2 fraction which then dissolved in a 0.01 m phosphate buffer solution of pH 7.0 containing 0.001 m CaCl_2_.

### PV-PLA2 activity analysis

The activity of PV-PLA2 was assessed using methods described by Marinetti based on its enzymatic activity on egg yolk [18]. The stages in this test begin with making an egg yolk suspension in a solution of 0.1 m Tris-HCl buffer pH 8.0. The egg yolk concentration in the solvent was 2 mg/ml. Then, 0.1 ml of the enzyme sample solution was reacted with 1.5 ml of egg yolk suspension. The decrease in absorbance value was observed using an ultraviolet-visible (UV-VIS) spectrophotometer at a wavelength of 900 nm for 5 min without the addition of enzyme samples.

Determination of protein concentration was performed by Lowry method (1951) using a biuret solution (1 ml of 1% CuSO_4_ solution and 1 ml of 1% NaK-tartrate solution in 100 ml of 2% Na_2_CO_3_ solution) and Folin–Ciocalteu Phenol 1N reagent. Standard curves were made using bovine serum albumin 200 μg/ml. The testing phase of protein concentration begins with adding the biuret solution into the sample; then, the sample was incubated for 10 min. The sample was then added with Folin reagent and then incubated again for 30 min. It was tested for absorbance values using a UV-VIS spectrophotometer at a wavelength of 750 nm.

Purity testing of protein was carried out using the sodium dodecyl sulfate-polyacrylamide gel electrophoresis (SDS-PAGE) method. The sample was dissolved in a buffer sample with a ratio of 1:2 and heated at 95°C for 1 min. Then, the gel was loaded into a gel mold tube and homogenized by flipping the tube. After the resolving gel solidifies, the stacking gel was prepared in the same way. After the gel and hole entering the sample were formed, then the gel was inserted into the electrophoresis chamber. The separation process was carried out at 20 mA until the dye reaches 0.5 cm at the bottom of the gel. The gel was then removed from the plate and soaked in a fixing buffer solution for 30 min followed by staining using a Staining Buffer solution. The gel was then rinsed using a destaining solution so that the protein can be visible in the gel.

### Antiretroviral activity analysis

Antiretroviral activity tests were performed in the culture of simian retrovirus-2-infected human cell line A549 cells (SRV2-A549). The test begins with a toxicity test against A549 cells with the 3-(4, 5-dimethyl thiazol-2yl)-2, 5-diphenyl tetrazolium bromide (MTT) assay method. The MTT assay was used to assess the cytotoxicity of PV-PLA2. SRV2-A549 cells (5×10^3^/well) were plated in 96-well plates; then, it was given sample extracts and incubated for 48 h at 5% CO_2_ and 37°C.

To obtained toxicity data, the sample extracts aliquoted and dropped into treatment wells subsequently; control, CV, and various amounts of PV-PLA2 (1, 2, 3, and 4 ppm). A549 cells grown into 96-well microplates with initial cell counts of 5000 cells/well were given sample extracts and incubated for 48 h at 5% CO_2_ and 37°C.

Tests were carried out for six types of samples, namely CV, results of precipitation of ET, results of ammonium sulfate immersion at 20% saturation level (AS20%), ammonium sulfate immersion results of 40% saturation level (AS40%), ammonium immersion sulfate saturation levels of 60% (AS60%), and ammonium sulfate immersion results of 80% saturation level (AS80%). Each sample was tested with four variations of concentration, namely 1, 2, 3, and 4 ppm in triploid. The samples were absorbed by a wavelength of 595 nm to determine the effect of samples on cells quantitatively. The absorbance value obtained is compared with the value of the absorbance of the control cell to determine the degree of inhibition of each sample.

Cultivation of SRV2-A549 culture was obtained by adding cultured cell (48×10^4^ cells/mL) on 12-well plates in Dulbecco’s Modified Eagle’s media, 5% fetal bovine serum, and 1% penicillin-streptomycin. It was added 500 μL of sample in each well for each type of sample at 5% CO_2_ and 37°C for 24 h. The culture was incubated for 7 days with the harvesting of supernatants carried out on days 1, 3, 5, and 7 as much as 500 µL which was then continued with the readministration of the same sample solution. Supernatant from harvesting is then stored in a refrigerator at −80°C to be used for quantitative real time-polymerase chain reaction (RT-qPCR).

RNA extraction aimed to obtain RNA of SRV-2 from SRV2-A549 culture which will be amplified and concentrated. QIAamp viral RNA Mini kit was used for RNA extraction from culture. RNA that has been successfully extracted was amplified and detected by RT-qPCR method. To be amplified by the PCR method, the extract of RNA which was obtained was first converted to cDNA using superscript TM III kit First-Strand Synthesis System for RT-PCR. Prepared samples was amplified by PCR method. The primary used in RT-qPCR was SRV-2 5737U19 and SRV-2 5943L20 (collection of PSSP IPB) which will amplify the gp70 gene. The PCR process was carried out using the iQ5 multicolor RT-PCR detection system. The PCR results were be read as the cycle threshold (Ct) value. The Ct value was used to measure the number of viruses in the sample. The SRV2 standard from concentrations of 10^1^-10^6^ was used as a reference for measuring the number of viruses.

## Results and Discussion

### PV-PLA2 activity

PV-PLA2 has obtained from the CV-PV purification. Its activity can be measured by Marinetti’s methods, Lowry’s test, and SDS-PAGE. The Marinetti methods using egg yolk based on its enzymatic activity to degrade PC. UV-Vis spectrophotometer at λ 900 nm was used to measure the enzyme activity. One enzyme unit is estimated as an activity that causes a reduction of 0.01 absorbance/min. As shown in Table-1, CV-PV exhibited less enzyme activity compared with others. Meanwhile, ammonium sulfate fractionation sample at 80% (AS80%) exhibited greater enzyme activity and made the egg yolk 1.81 times more pure than CV-PV did.

**Table-1:** Result of PV-PLA2 activity test with Marinetti’s and Lowry’s methods.

Sample	Protein concentration	Vol	Enzyme unit	Enzyme activity	Total activity	Total protein	Specific activity	Purity
						
	µg/mL	mL	Unit	Unit/mL	Unit	µg	Unit/µg	
CV	330.81	4	0.5	5	20	1323.22	0.015	1
ET	310.61	4	0.36	3.6	14.4	1242.44	0.012	0.77
AS20	65.28	4	0.48	4.8	19.2	261.11	0.074	4.86
AS40	318.22	4	0.2	2	8	1272.89	0.006	0.42
AS60	289.00	4	0.26	2.6	10.4	1156.00	0.009	0.60
AS80	263.67	4	0.72	7.2	28.8	1054.67	0.027	1.81

PV-PLA2=*Pterois* *volitans* phospholipase A2, CV=Crude venom, ET=Ethanol, AS20%=Ammonium sulfate immersion at 20% saturation level, AS40%=Ammonium sulfate immersion at 40% saturation level, AS60%=Ammonium sulfate immersion at 60% saturation level, AS80%=Ammonium sulfate immersion at 80% saturation level

According to the Lowry test results in Table-1, it was shown that protein concentration tended to decrease after going through the purification stages. CV-PV exhibited a greater initial protein concentration compared with others. Meanwhile, AS80% exhibited less initial protein concentration (263.67 μg/mL). The purer the protein concentration, the higher the PV-PLA2 activity.

**Figure-1:**
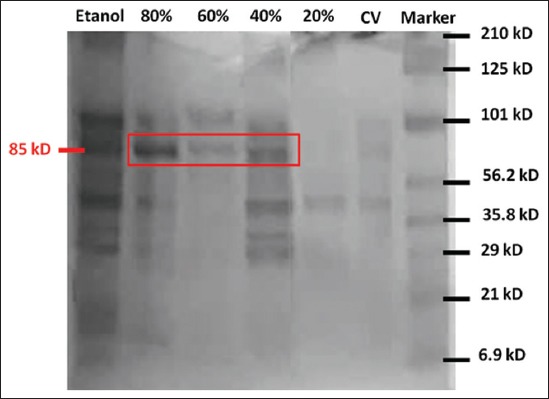
The result of *Pterois volitans* phospholipase A2 activity test with sulfate-polyacrylamide gel electrophoresis.

SDS-PAGE was carried out to control the quality of PV-PLA2 and determination of its protein profile. The venom samples from PV were loaded onto a 12% polyacrylamide gel. SDS-PAGE results showed eight separate bands in the gel. The molecular weight of observed protein ranged from 6.9 to 210 kDa (Figure-1). This result is approximately in accordance with PV venom obtained by Choromanski which ranged from 29 to 205 kDa and other *Pterois* species, *P. russelii* venom, which ranged from 6 to 205 kDa [19,20]. Based on the results in Figure-1 of the SDS-PAGE test, it can be seen that the isolation of PV-PLA2 using AS80% exhibits the highest level of purity (85 kDa) compared to the standard. There was an increase in purity as the salting stage progresses - the PV-PLA2 isolation. It related to the result of Savitri *et al*. that obtained PLA2 isolation from the spines venom of the crown-of-thorns starfish [21].

### Toxicity test

There are three tests carried out for antiretroviral activity analysis: MTT assay test, the SRV2 antiretroviral activity test, and the RT-qPCR test. MTT assay test is used to determine the level of toxicity of the samples tested on human cells. The A549 cells were used because these cells have several characteristics of human cells such as the ability to synthesize phospholipids, lamellar cytoplasmic bodies, and apical microvillus [22]. Furthermore, morphologically, A549 cells in the form of epithelium are similar in shape to human living cells [23]. Hence, it is expected that its use can illustrate the original effect of the sample being tested on human cells.

**Figure-2:**
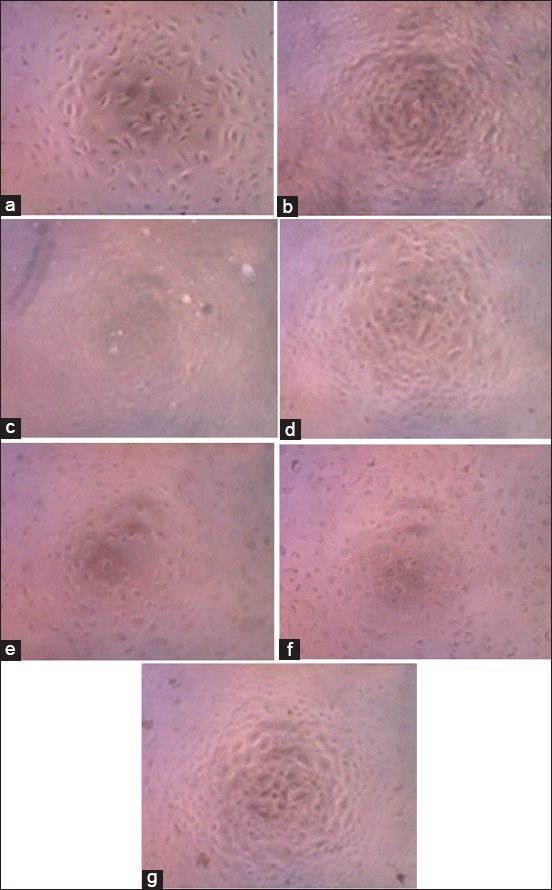
A549 cell morphology in concentration 4 ppm for sample: (a) Control; (b) crude venom; (c) ethanol; (d) ammonium sulfate immersion at 20% saturation level; (e) ammonium sulfate immersion at 40% saturation level; (f) ammonium sulfate immersion at 60% saturation level; (g) ammonium sulfate immersion at 80% saturation level.

Tests were carried out for six types of samples, namely CV, ET, AS20, AS40, AS60, and AS80 (Figure-2). Each sample was tested with four variations of concentration, namely 1, 2, 3, and 4 ppm triploid. The samples were analyzed on the wavelength of 595 nm for absorbance to determine the effect of samples on cells quantitatively. The absorbance value obtained is compared with the value of the absorbance of the control cell to determine the degree of inhibition of each sample.

After the addition of 1-4 ppm samples in Table-2, the lethal concentration 50 (LC_50_) of each sample can be calculated. Based on the results of the calculation, it was found that several samples had a degree of inhibition >50%. This indicates that the rate of cell death was very high. The sample was very toxic and not suitable for use in subsequent tests. Addition of 1 mg/ml AS80% resulted in a cell death rate of approximately 8.81%. The lower the concentration of toxin added, the less cell death occurred in the SRV-2 culture; this can be expressed as y=0.0499x+0.0086. Probit analysis was conducted to determine LC_50_, which was 9.85 mg/ml. This is the maximum dosage tolerated by the SRV-2. Therefore, the concentration of PV-PLA2 must be below this value for >50% of cells to survive. This is a lower value than the LC_50_ of PLA2 from *A. planci* venom obtained by Wijanarko *et al*. which was 1.64 mg/ml [10].

**Table-2:** The result of toxicity test with MTT assay.

Sample	Inhibition degree (%)			

	4 ppm	3 ppm	2 ppm	1 ppm
CV	−101	−153	−164	−176
ET	1	−8	−12	−27
AS20	10	1	1	−9
AS40	67	56	55	52
AS60	71	71	61	57
AS80	21	16	10	6

MTT=3-(4, 5-dimethyl thiazol-2yl)-2, 5-diphenyl tetrazolium bromide, CV=Crude venom, ET=Ethanol, AS20%=Ammonium sulfate immersion at 20% saturation level, AS40%=Ammonium sulfate immersion at 40% saturation level, AS60%=Ammonium sulfate immersion at 60% saturation level, AS80%=Ammonium sulfate immersion at 80% saturation level

There are four safe samples to be used in the next test stages, namely CV, ET, AS20, and AS80 in each variation of concentration. However, when compared with the test results in the previous stage, ET and AS20 have a smaller purity compared to AS80. Both samples are considered not feasible to be tested because there is no improvement in quality despite being given additional treatment. So both samples are not economically suitable. Therefore, the sample that will be continued to be tested for antiretroviral activity is the CV and AS80 sample.

### Analysis of antiretroviral activity

In the test phase of antiretroviral SRV-2 activity, SRV2-A549 cells were cultured inside 24 wells to test two types of samples with four variations of concentration in duplicate. Each sample was harvested at 7 days of incubation to ensure that both SRV2 grew. Each harvested samples then being tested in RT-qPCR to identify the copy number of SRV2. The results of PV-PLA2 antiretroviral activity were shown in RT-PCR results in Table-3. *In vitro*, PV-PLA2 has antiretroviral activity against SRV2, with 97% inhibition rate for purification results with AS80.

**Table-3:** Analysis of antiretroviral activity with RT-qPCR.

No.	Sample	Copy number	Percentage of inhibition (%)
1	80% 1 ppm	764,539	79.87
2	80% 2 ppm	577,150	84.80
3	80% 3 ppm	101,168	97.34
4	80% 4 ppm	84,359	97.78
5	Negative control	3,797,255	0.00
6	CV 1 ppm	1,737,825	54.23
7	CV 2 ppm	1,624,415	57.22
8	CV 3 ppm	605,334	84.06
9	CV 4 ppm	600,111	84.20
10	Positive control	17,183	99.55

CV=Crude venom, RT-qPCR=Quantitative real time-polymerase chain reaction

This research has proved that the bioactive components of animal venoms are divulging the anti-HIV activities. In this context, the article discloses the decisive role of animal venoms as alternative natural resources to treat HIV/AIDS infection and propound the plausible development of new therapeutic drugs in the 21^st^-century era [24]. In previous research, antimicrobial peptides (AMPs) from fish AMPs have great implications beyond the fight against pathogens [25]. Drug development from aquaculture animal venoms might create a new therapeutic era and their future applications in both the aquaculture industry and human health care.

The finding of this research supports the hypothesis of PLA2 from animal venom which exhibits antiviral activity that may inhibit HIV replication by several other researchers [8,10,26]. Our results are similar to Fenard *et al*. who obtained that anti-HIV activity of PLA2 isolated from bee and snake venoms among their biological effects interacts with the host cells and prevents the intracellular release of HIV capsid protein, suggesting that they block viral entry into the cells before virion uncoating [8]. Then, PLA2 isolated from *A. planci* venom has the potential to develop as an anti-HIV because *in vitro* experiment showed its ability to decrease HIV infection in peripheral blood mononuclear cells and the number of HIV ribonucleic acid in culture [10]. Villarrubia *et al*. found that PLA2-Cdt from *Crotalus durissus* terrific have anti-HIV activity with Gag p24 processing inhibition mechanism [26]. ART from venomous animal such as active constituents of bee, scorpion, and snake venom is promising in the fight against HIV because it has the potential to reduce the passage of HIV-1 and to limit the viral load in infected people [27]. PV-PLA2 is a promising source of a potentially cheap and environmentally friendly anti-HIV agent that could potentially provide an effective solution in treating one of the most dangerous diseases affecting humanity today.

## Conclusion

Methods of precipitation of protein using ammonium sulfate fractionation at 80% saturation level more effective purify PV-PLA2 with a high level of purity compared to precipitation of protein using ET. In the present study, PV-PLA2 was produced from CV by a simple extraction process. PV-PLA2 has been shown antiretroviral activity against simian retrovirus serotype-2 *in vitro*, with 97% inhibition rate for AS80% samples. The significant inhibition of SRV2-A549 culture shown that the PV-PLA2 extracted from PV venom has the potential to become anti-HIV substances. It would be worthwhile to further evaluate the antiretroviral activity of PV-PLA2 in the *in vivo* studies. PV-PLA2 is a promising source of a potentially cheap and environmentally friendly anti-HIV agent that could potentially provide an effective solution in treating one of the most dangerous diseases affecting humanity today.

## Authors’ Contributions

ANS, AW, and MS: Arranged, designed, and supervised the study. MYA: Carried out sampling and laboratory analysis and wrote the first draft of the manuscript. MJG and RMYA: Analyzed the data. ANS, AW, DKP, and MS: Contributed to the writing of the manuscript. ANS, AW, HH, DKP, and MS: Jointly developed the structure and arguments for the paper. DKP, HH, and MJG: Made critical revisions. All authors read and approved the final manuscript.
